# The bHLH Transcription Factor Hand Regulates the Expression of Genes Critical to Heart and Muscle Function in *Drosophila melanogaster*

**DOI:** 10.1371/journal.pone.0134204

**Published:** 2015-08-07

**Authors:** Benjamin Hallier, Julia Hoffmann, Thomas Roeder, Markus Tögel, Heiko Meyer, Achim Paululat

**Affiliations:** 1 Department of Zoology/Developmental Biology, University of Osnabrück, 49069 Osnabrück, Germany; 2 Department of Animal Physiology, University of Kiel, 24098 Kiel, Germany; 3 Weatherall Institute of Molecular Medicine, University of Oxford, OX3 9DS Oxford, United Kingdom; Oxford Brookes University, UNITED KINGDOM

## Abstract

Hand proteins belong to the highly conserved family of basic Helix-Loop-Helix transcription factors and are critical to distinct developmental processes, including cardiogenesis and neurogenesis in vertebrates. In *Drosophila melanogaster* a single orthologous *hand* gene is expressed with absence of the respective protein causing semilethality during early larval instars. Surviving adult animals suffer from shortened lifespan associated with a disorganized myofibrillar structure being apparent in the dorsal vessel, the wing hearts and in midgut tissue. Based on these data, the major biological significance of Hand seems to be related to muscle development, maintenance or function; however, up to now the physiological basis for Hand functionality remains elusive. Thus, the identification of genes whose expression is, directly or indirectly, regulated by Hand has considerable relevance with respect to understanding its biological functionality in flies and vertebrates. Beneficially, *hand* mutants are viable and exhibit affected tissues, which renders *Drosophila* an ideal model to investigate up- or downregulated target genes by a comparative microarray approach focusing on the respective tissues from mutant specimens. Our present work reveals for the first time that *Drosophila* Hand regulates the expression of numerous genes of diverse physiological relevancy, including distinct factors required for proper muscle development and function such as Zasp52 or Msp-300. These results relate Hand activity to muscle integrity and functionality and may thus be highly beneficial to the evaluation of corresponding *hand* phenotypes.

## Introduction

Basic helix-loop-helix (bHLH) transcription factors are key regulators of numerous developmental processes including cardiovascular development, hematopoiesis, stem cell maintenance, neurogenesis and myogenesis (reviewed by [1]). Among the class II bHLH family, Hand proteins constitute a prominent class regulating, e.g., trophoblast development, limb bud outgrowth, branchial arch development or cardiogenesis [2–14]. In higher vertebrates, e.g. mouse or human, two paralogous *hand* genes (*hand1*, *hand2*) are present in the genome, with both being expressed in the lateral plate mesoderm, distinct neural crest cells and the developing heart. With respect to the latter tissue, expression of both genes is initially overlapping. Upon formation of a linear heart tube however, *hand1* expression becomes restricted to the developing left ventricle, while *hand2* is still expressed throughout the complete heart primordium. As soon as cardiac looping initiates, *hand2* expression becomes restricted to the heart region the right ventricle arises from [5, 15, 16]. Thus, the two paralogous *hand* genes in higher vertebrates may have evolved different tissue specific functionalities during cardiogenesis. A critical function of Hand in cardiogenesis was demonstrated by analyzing the phenotypes of loss of function mutations in mice where severe malformations of the heart were observed in animals lacking Hand activity [5, 6, 17]. For instance, mice being mutant for both, *hand1* and *hand2* are embryonic lethal and, among other cardiac defects, characterized by severe ventricular hypoplasia [18]. In addition, Natarahan and colleagues reported that *hand1* expression is almost absent in hearts of patients suffering from ischemic or dilated cardiomyopathy, whereas *hand2* expression remains unchanged [19].

In *Drosophila*, only a single Hand orthologue was discovered by extensive genome-wide searches for bHLH sequences [20]. Notably, Hand represents the only transcription factor identified so far that is expressed in all three major embryonic cell types that comprise the *Drosophila* circulatory system (cardioblasts, pericardial cells, and hematopoietic progenitors in the lymph gland). In addition to these cells, *Drosophila hand* is expressed in the circular visceral musculature and in distinct cells belonging to the central nervous system [21–25]. Despite its expression in the embryonic tissues mentioned above, Hand knock-out phenotypes manifest primarily in the dorsal vessel and the gut of adult animals, indicating an essential role of Hand in the remodeling of these tissues during metamorphosis rather than being required for proper determination and differentiation of the respective tissues during embryogenesis. Apparently, the phenotypes are mainly characterized by an abnormal arrangement of muscle fibers in the corresponding tissues [24]. This observation together with recent data that also describe severe disarrangements in muscle cells of *hand* mutant wing hearts [26] indicates that lack of Hand activity predominantly affects muscle structure or integrity.

Here we took advantage of the fact that the *Drosophila* genome carries only a single *hand* orthologue and that homozygous *hand* mutant individuals survive into adulthood at significant rates. Cardiogenesis initially proceeds normally in such animals, however, the mature heart displays several structural malformations including sarcomere defects, which may account, together with other phenotypes manifesting in gut morphogenesis and wing heart development, for a shortened lifespan and reduced fitness. The facts depicted above render *Drosophila* an ideal model to identify genes regulated by Hand, which may also promote a detailed understanding of *hand* functionality in vertebrates. For instance, downregulation of human *hand1* in cardiomyopathies, as observed by [19], may result in misregulation of yet unknown target genes in the heart and thereby lead to an age-dependent aggravation of cardiac pathologies.

Herein we present a genome wide microarray based approach to identify genes that are, directly or indirectly, regulated by Hand. As a result we identified several genes that exhibit a considerably up- or downregulated expression in *hand* mutant animals, compared to wild type. Due to the Hand knock-out phenotypes depicted above, in subsequent experiments we focused primarily on genes that are involved in muscle cell development, muscle structure maintenance or muscle physiology. With respect to genes matching these criteria we did quantitative real-time PCRs as well as quantitative Northern blots in order to validate the corresponding microarray data, thus minimizing the possibility of considering false positives. In sum, our data demonstrate for the first time that *Drosophila* Hand is regulating the expression of several muscle specific proteins and may thus be highly beneficial for understanding distinct *hand* null phenotypes described earlier. Furthermore, the catalogue of genes apparently regulated by Hand provides a solid basis for future investigations on the regulatory networks that are crucial to establishing heart and muscle architecture or functionality.

## Materials and Methods

### Fly strains

w1118 was used as wild type control. The *hand* ^*173*^ null mutant was kindly provided by Manfred Frasch [24].

### Heart preparations

For optimal growth conditions, wild type (w1118) and homozygous mutant (*hand* ^*173*^) flies were raised at low population density on standard medium at 22°C. Hearts were dissected from wandering 3^rd^ instar larvae since the corresponding animals represent a clearly defined developmental stage. In addition, *hand* ^*173*^ 3^rd^ instar larvae do neither exhibit increased lethality rates [23], nor any developmental delay (own observation), compared to wild type. By selecting such animals we minimize the possibility of analyzing dying animals, which may be characterized by a considerably altered transcript composition, compared to healthy specimen. Wandering 3^rd^ instar larvae were collected, transferred into a tea basket and anesthetized by incubating the larvae at 60°C for 15 seconds in a water bath. Such treatment induces stretching of the larvae with a relaxed muscle contraction state, which facilitates the subsequent heart preparation. All further dissections were carried out in PBS. Firstly, individual larvae were pinned upside down to sylgard plates with preparation needles. The most anterior and posterior portion of each larvae was removed with micro-scissors. Next, larvae were opened from the ventral side and all viscera were carefully removed to allow direct access to the heart. About 100 heart tubes including associated tissue (pericardial cells, alary muscles) from both genotypes were carefully extracted and directly collected in Trizol (Invitrogen—Life Technologies, Frankfurt, Germany) for subsequent RNA preparations.

### Microarray

Microarray analyses were essentially performed as described earlier [27, 28]. In brief, cDNA synthesis from RNA isolated from manually dissected larval hearts was performed with Prime Script RT (Takara, Saint-Germain-en-Laye, France) using the following primers: OdT T7 I (5’-GAG AGA GGA TCC AAG TAC TAA TAC GAC TCA CTA TAG GGA GAT TTT TTT TTT TTT TTT TTT TTT T G/A/C-3’) and CapFinder Sp6rG (5’-CAG CGG CCG CAG ATT TAG GTG ACA CTA TAG A rGrGrG-3’). cDNA was amplified with OdT T7 II (5’-GAG AGA GGA TCC AAG TAC TAA TAC GAC TCA CTA TAG G-3’), Adaptor Sp6rG (5’-GAC GCC TGC AGG CGA TGA ATT TAG G-3’) and LA Taq polymerase [29]. cDNA was transcribed with MEGAscript T7 including aminoallyl-UTP (Ambion—Life Technologies, Frankfurt, Germany) and subsequently labeled with Alexa Fluor 555 or 647 (Invitrogen—Life Technologies) for control or experimental sample, respectively. Samples were hybridized to *Drosophila* 14 k V2 DNA-microarrays (Canadian *Drosophila* Microarray Centre, Toronto, Canada) and scanned using a GenePix 4000B Microarray Scanner (Axon Instruments, Molecular Devices, Biberach, Germany). Data acquisition, normalization and analysis including hierarchical clustering were carried out with the programs GenePix 6.0 and Acuity 4.1 (Axon Instruments, Molecular Devices, Biberach, Germany). We performed two channel probe hybridizations with three technical replicates. Genes with a more than 1.33 fold increased transcript level in *hand* deficient samples, compared to matching controls in at least two out of three technical replicates, were scored as upregulated; those with a less than 0.67 fold relative transcript level in at least two out of three technical replicates were scored as downregulated. In order to minimize the impact of possible variations between individual specimens, wild type and mutant RNA, respectively, was extracted from a high number of dissected heart tubes (>100). The corresponding individual preparations were pooled according to their genotype and further analyzed as depicted above. Due to the fact that 2–3 values per gene do not allow for a reliable statistical analysis we did not calculate p-values that would probably not reflect the real data situation. Instead of that we extracted the most relevant genes from these analyses and quantified their differential expression with alternative methods (see results).

The DNA-microarray experiments have been deposited in the GEO-database (accession number GSE64429).

### qRT-PCR

Total-RNA isolated from complete wandering stage 3rd instar larvae (RNeasy Mini Kit, Qiagen, Hilden, Germany) was treated with DNase I (Invitrogen—Life Technologies) according to the manufacturer’s instructions and used as a template for cDNA synthesis (SuperScript III Reverse Transcriptase, Invitrogen—Life Technologies). qRT-PCR was conducted according to standard protocols using DyNAmo ColorFlash SYBR Green qPCR Kit (Biozym, Hessisch Oldendorf, Germany) and an iCycler iQ Real-Time PCR System (Bio-Rad, Munich, Germany). Primer pairs were designed with QuantPrime [30] applying the presettings to consider only regions containing at least one intron and to accept splice variant hits. Data were evaluated as described in [31]. Genes with an at least 1.33 fold increased transcript level in *hand* deficient samples, compared to controls, were scored as upregulated, those with a less than 0.67 fold relative transcript level were scored as downregulated. Primers used are summarized in S2 Table. For each gene at least three biological replicates were performed.

### Northern blot and riboprobe synthesis

Northern blots were conducted as described [32] using total RNA (15μg per lane) isolated from complete wandering stage 3^rd^ instar larvae and a hybridization temperature of 66°C. Quantification of the relative band intensities in relation to the loading controls (ribosomal RNA) was done by densitometric analysis using a VersaDoc 4000 imaging system (Bio-Rad Laboratories, Hercules, CA, U.S.A.) and Quantity One software, version 4.6.9.

Templates for riboprobe synthesis were generated using the following primer pairs:

*kugelei*: atgaagattaaaaaatatgta (forward, FW), gacattctcaaaaatgggatc (reverse, RV); *msp-300*: gcgcgataaggagcaacaggt (FW), cctgttgctcggctaatgcgc (RV); *zasp52*: atggcccaaccacagctgctg (FW), gctgttgctgctgctatagtt (RV); *cubitus interruptus*: atggacgcctacgcgttacc (FW), agccttcaaacgtgcatttgt (RV); *hedgehog*: atggataaccacagctcagtg (FW), tcaatcgtggcgccagctctg (RV); *mef2*: atgggccgcaaaaaaattcaa (FW), ctatgtgcccaatccgcccga (RV)

Probes were synthesized by *in vitro* transcription using “DIG RNA labeling kit” (Roche, Mannheim, Germany). For each gene at least three biological replicates were performed.

## Results

### The expression of numerous genes is altered in hand mutant hearts

**Fig 1:**
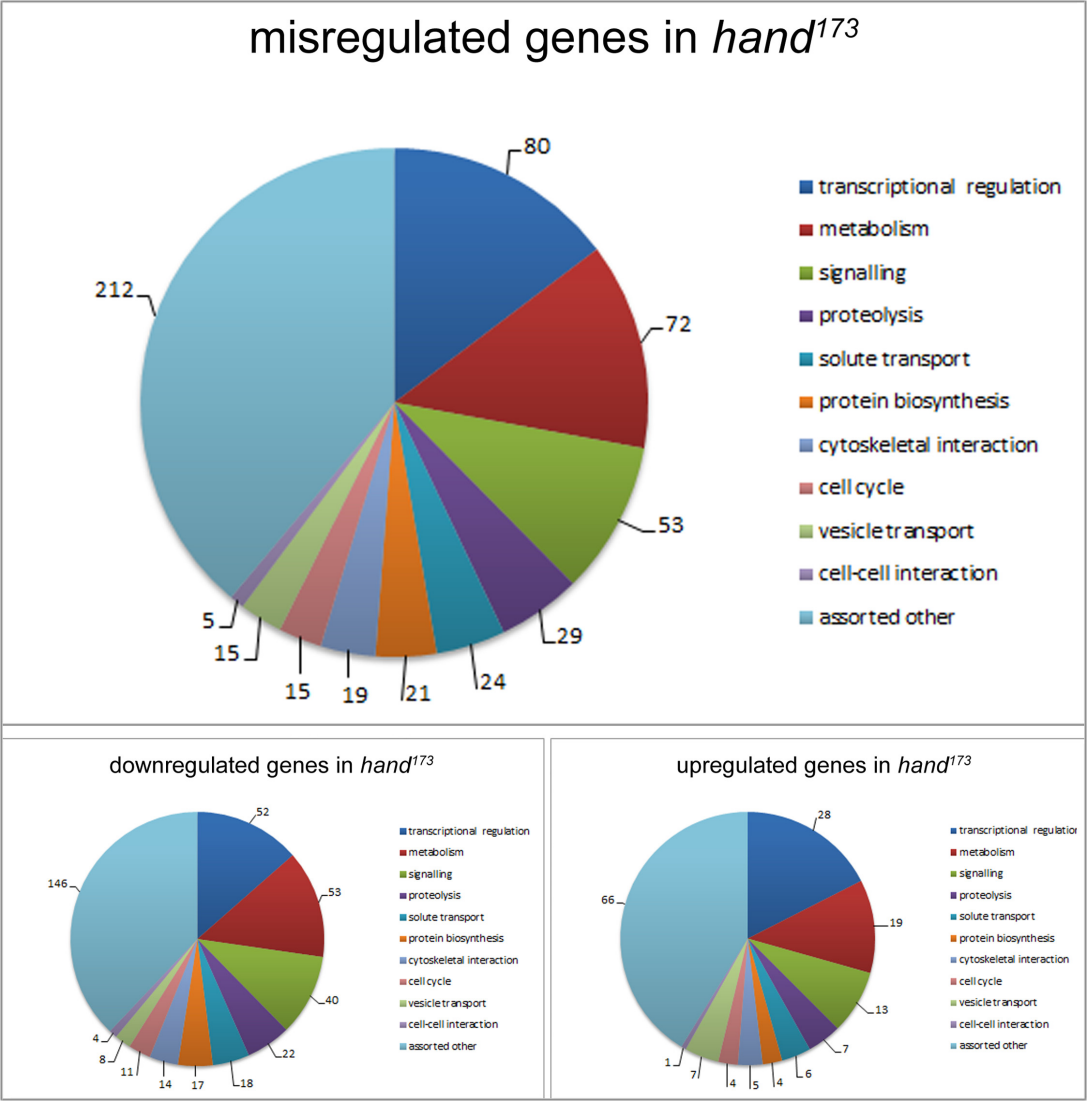
Functional classification of genes exhibiting deviant expression levels in *hand* ^*173*^ animals. *Drosophila* Hand is apparently involved in regulating the expression of 545 genes with 385 genes being downregulated in *hand* mutants (*hand* ^*173*^) and 160 genes displaying an increased expression in the same line, compared to wild type. Functional classification of the corresponding protein products was done manually utilizing data from NCBI (http://www.ncbi.nlm.nih.gov/) and Flybase (http://flybase.org/). Factors with yet unknown physiological functions are allocated to the category “assorted other”.

In previous studies it was shown that *Drosophila* Hand functions as a potent transcriptional activator [23] that is essential for proper morphogenesis of adult heart and midgut tissue [24] but also for wing heart differentiation [26]. However, the target genes of the transcription factor remained elusive up to now. In order to identify genes whose expression is regulated by Hand, we did genome wide microarrays using total RNA isolated from dissected 3^rd^ instar larval hearts of wild type as well as *hand* null animals (*hand* ^*173*^, [24]) and compared the expression levels of the complete set of genes in the respective genetic backgrounds. In order to minimize the impact of variations between individual specimens, we extracted and pooled RNA from a high number of individual heart preparations instead of doing biological replicates with less hearts. Noteworthy, the semilethal phase of *hand* mutant animals is during 1^st^ and 2^nd^ larval instars with apparently no 3^rd^ larval instar lethality [24]. Thus, the possibility of analyzing dying animals that may exhibit an abnormal gene expression can be excluded. Isolated hearts were selected since they represent the major Hand expressing tissue in *Drosophila* [21]. As shown in Fig 1, analysis of the microarray data yielded 545 genes exhibiting an altered expression in *hand* ^*173*^ hearts, with 385 genes being downregulated in the mutant and 160 genes displaying an increased expression in the same line, compared to wild type. The fact that the number of downregulated genes exceeds that of activated genes by a factor of more than two indicates that Hand acts predominantly as a transcriptional activator rather than being a repressor in larval hearts. Noteworthy, the in principle capability of *Drosophila* Hand to act as a potent transcriptional activator has been shown previously, however, the respective study did not yield any *in vivo* target of the protein [23]. A functional classification of the protein products corresponding to the genes identified by the microarray revealed highly diverse physiological functions of the respective factors, with proteins involved in transcriptional regulation being most abundant (80 genes). Consistent with a high demand for energy in heart tissue, genes participating in metabolism are also enriched (72). As a third prominent category, signaling factors are abound (53). Other noteworthy classes include genes with protein products being involved in proteolysis (29), solute transport (24), protein biosynthesis (21), and cytoskeletal interactions (19). The remaining functional categories comprise genes involved in the progression of the cell cycle (15), vesicle transport (15), and cell-to-cell interactions (5). A last category contains “assorted other” genes that are either of unknown function or do not fit into any of the former categories (212). Individual genes within each group are depicted in S1 Table.

### Hand regulates the expression of genes critical to muscle and heart development or function

Despite the high number of misregulated genes in *hand* ^*173*^ animals (Fig 1), only mild phenotypes were observed in this line with the majority of them being related to impaired muscle and heart structure or integrity [24, 26]. Thus, the major biological significance of Hand is apparently related to muscle and heart development, maintenance or function. However, up to now the physiological basis for this functionality remained elusive. In order to analyze this issue in more detail we screened the microarray data for proteins that are known to be essential for these processes. In addition, we concentrated on genes that were confirmed to be expressed in pupal [33] or adult hearts [34] and on genes that were shown to be critical to heart functionality [35]. By applying these parameters we narrowed down the number of potentially most relevant target genes to 42, with 2 of these genes being upregulated in *hand* ^*173*^ and 40 displaying a reduced expression in the same line, compared to wild type (Table 1).

**Table 1:** Preliminary selection of genes exhibiting deviant expression levels in *hand* ^*173*^ animals. By screening the microarray dataset for factors that are either significantly expressed in heart tissue or involved in muscle or heart development, maintenance or function, we identified 42 genes of potentially high physiological relevance. While 2 of these genes are upregulated in *hand* ^*173*^, 40 display a reduced expression in the same line, compared to wild type. Functional classification of the corresponding protein products was done manually utilizing data from NCBI (http://www.ncbi.nlm.nih.gov/) and Flybase (http://flybase.org/). Factors with yet unknown physiological functions are allocated to the category “assorted other”.

#	gene	name	expression in *hand* ^*173*^	functional category	#	gene	name	expression in *hand* ^*173*^	functional category
1	CG1161	*-*	down	assorted other	22	CG10679	*nedd8*	down	proteolysis
2	CG1520	*wasp*	down	cytoskeletal interaction	23	CG10811	*eukaryotic translation initiation factor 4G*	down	cell cycle
3	CG1844	*selenoprotein G*	down	assorted other	24	CG11271	*ribosomal protein S12*	down	protein biosynthesis
4	CG2145	*-*	down	proteolysis	25	CG11525	*cyclin G*	down	cell cycle
5	CG2233	*-*	down	assorted other	26	CG11661	*neural conserved at 73EF*	down	metabolism
6	CG3186	*eIF-5A*	down	protein biosynthesis	27	CG12400	*NADH dehydrogenase (ubiquinone) B14*.*5 B subunit*	down	metabolism
7	CG3869	*mitochondrial assembly regulatory factor*	down	signaling	28	CG14035	*msp-300*	down	cytoskeletal interaction
8	CG4699	*waharan*	down	cell cycle	29	CG14616	*lethal (1) G0196*	up	metabolism
9	CG4716	*methylenetetrahydrofolate dehydrogenase [NAD(P)+] activity*	down	metabolism	30	CG15067	*-*	down	assorted other
10	CG5277	*intronic protein 259*	down	assorted other	31	CG16713	*-*	down	proteolysis
11	CG5320	*glutamate dehydrogenase*	up	metabolism	32	CG16747	*ornithine decarboxylase antizyme*	down	metabolism
12	CG5399	*-*	down	assorted other	33	CG17108	*-*	down	assorted other
13	CG6090	*ribosomal protein L34a*	down	protein biosynthesis	34		* *		
14	CG6105	*-*	down	metabolism	35	CG17820	*female-specific independent of transformer*	down	assorted other
15	CG6746	*-*	down	signaling	36	CG18039	*kaiRIA*	down	signaling
16	CG7749	*kugelei*	down	cytoskeletal interaction	37	CG18107	*-*	down	assorted other
17	CG8226	*translocase of outer membrane 7*	down	solute transport	38	CG30084	*zasp52*	down	cytoskeletal interaction
18	CG8369	*-*	down	assorted other	39	CG30415	*-*	down	assorted other
19	CG8580	*akirin*	down	transcriptional regulation	40	CG31509	*turandot A*	down	assorted other
20	CG9470	*metallothionein A*	down	metabolism	41	CG33171	*multiplexin*	down	cytoskeletal interaction
21	CG10484	*regulatory particle non-ATPase 3*	down	proteolysis	42	CG33256	*limpet*	down	transcriptional regulation

**Fig 2:**
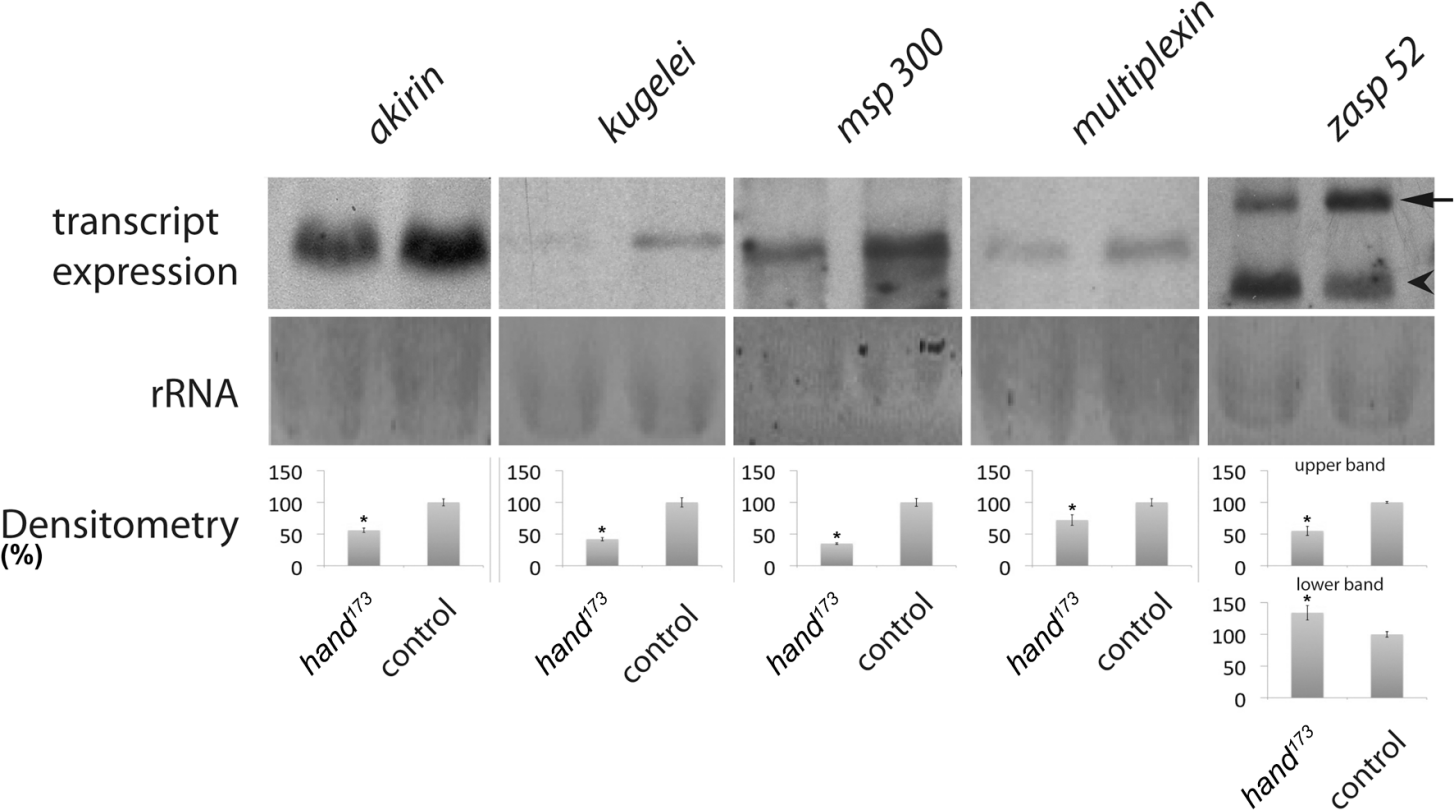
Relative expression of selected genes in hand mutant animals, compared to wild type. Expression levels were assessed by Northern blot. Statistically relevant deviations are evident with respect to all genes tested. While *akirin* displays an expression that is reduced by 40.5% in the *hand* mutant (*hand* ^*173*^), the expression of *kugelei* is lowered by 57.5%, that of *msp-300* by 65% and that of *multiplexin* by 28.5%, respectively. With regard to *zasp52*, two major transcripts are detected by the applied riboprobes with the larger one (arrow) being downregulated by 44.8% in the *hand* mutant and the smaller one (arrowhead) being upregulated by 34.9% in the same line. Bars represent mean values + SD of at least three independent experiments. Asterisks indicate statistical significance (Student´s *t*-test *P*<0.05). Quantification of the relative band intensities in relation to the loading controls (ribosomal RNA, rRNA) was done by densitometric analysis.

In order to validate the corresponding microarray data we re-analyzed the expression levels of the selected 42 genes by quantitative real time-PCR (qRT-PCR). To monitor the overall changes in gene expression, irrespective of a possible isoform specific regulation, primers detecting multiple splice variants were generated. The corresponding sequences are depicted in S2 Table. In individual cases, the qRT-PCR data were further validated by Northern blot analyses (see below). Due to the fact that especially the latter technique requires high amounts of RNA, we isolated total RNA from complete 3^rd^ instar larvae instead of extracting it from dissected heart tissue. Since, in addition to the heart, only few other tissues are expressing Hand [21], we consider the comparability of these data with the microarray derived results as being acceptable. Furthermore, by isolating RNA from complete 3^rd^ instar larvae, we minimize the possibility of systemic errors that may arise in the course of nucleotide amplification, which was a mandatory experimental step in order to obtain sufficient sample from heart tissue to conduct the microarrays (see materials and methods). By applying the corresponding non-amplified cDNA preparations in qRT-PCR analyses, we confirmed the microarray results with respect to 13 genes (Table 2), while 25 genes showed expression aberrations that were either below the minimal threshold for provisional acceptance or not congruent with the microarray data. The expression of four additional genes, *akirin*, *kugelei*, *multiplexin*, and *zasp52*, respectively, did not exhibit a statistically significant deviation in *hand* ^*173*^ animals. However, due to the high functional relevance of the corresponding protein products to muscle functionality [36–39], as well as to address the discrepancy between microarray and qRT-PCR data, we decided to list the respective genes in Table 2 and re-analyze their expression in *hand* ^*173*^ animals by applying Northern blot as a third method. This methodology is considered highly convenient for this issue since it provides a direct relative comparison of transcript abundance between the individual samples. As a positive control we also re-analyzed the expression of *msp-300* in wild type as well as *hand* mutant animals. As depicted in Fig 2, the comparative Northern blots proved a significant misregulation of all corresponding genes in *hand* mutant animals, thus confirming the microarray data and validating a Hand dependent expression of the respective factors: while *akirin* displays an expression that is reduced by 40.5% in the *hand* mutant, the expression of *kugelei* is lowered by 57.5%, that of *msp-300* by 65% and that of *multiplexin* by 28.5%, respectively. With regard to *zasp52*, two major transcripts are detected by the applied riboprobes with the larger one being downregulated by 44.8% in the *hand* mutant and the smaller one being upregulated by 34.9%. The latter result indicates a splice variant specific regulation of the corresponding gene, which has already been described [40] and which may account for the inconsistent qRT-PCR result that presumably reflects the simultaneous up- as well as downregulation of individual splice variants. Since the primers applied in the respective qRT-PCR analyses are specific to 11 (C, F, I, K, L, M, R, S, T, U, W) of the 18 predicted *zasp52* splice variants (http://flybase.org), the individual changes in expression of these 11 transcripts may compensate for each other, thus resulting in an overall insignificant change in expression as determined by qRT-PCR (Table 2). By discriminating between individual splice variants the Northern blot data depicted in Fig 2 clearly confirm a Hand dependent expression of *zasp52*. Based on the apparent molecular masses of the detected transcripts, the larger one consists of about 4.7 kilobases, while the smaller one comprises about 3.1 kilobases (S1 Fig).

**Table 2:** Genes exhibiting deviant expression levels in *hand* ^*173*^ animals, as confirmed by qRT-PCR. Genes listed are either expressed in heart tissue or involved in muscle and heart development, maintenance or function. Deviating expression of the particular genes in *hand* ^*173*^ animals is depicted in percent (%) relative to the respective expression in wild type specimen, which was set to 100%. Statistically significant deviations are indicated (* *P*<0.05; ** *P*<0.01, Student´s *t*-test). Functional classification of the corresponding protein products was done manually utilizing data from NCBI (http://www.ncbi.nlm.nih.gov/) and Flybase (http://flybase.org/). Values represent means of at least three independent qRT-PCR experiments.

#	gene	name	expression in *hand* ^*173*^ [%]	function / biological process
1	CG10484	*regulatory particle non-ATPase 3*	-38	protein degradation
2	CG11661	*neural conserved at 73EF*	-74,2	tricarboxylic acid cycle
3	CG12400	*NADH dehydrogenase (ubiquinone) B14*.*5 B subunit*	-51,9	mitochondrial electron transport
4	CG14035	*muscle-specific protein 300 (msp-300)*	-77	cellular component organization
5	CG15067	*-*	-73,5	neurogenesis
6	CG16747	*ornithine decarboxy-lase antizyme*	-42,6	cell differentiation
7	CG17108	*-*	-68	unknown
8	CG18107	*-*	-51	unknown
9	CG2145	*-*	-65,6	peptide degradation
10	CG2233	*-*	-69,2	unknown
11	CG30084	*zasp52*	-51	muscle structure development
12	CG30415	*-*	-53,9	unknown
13	CG33171	*multiplexin*	-33	cell adhesion
14	CG3869	*mitochondrial assembly regulatory factor (marf)*	-55,1	mitochondrion organization
15	CG7749	*kugelei*	-61	cellular component organization
16	CG8580	*akirin*	-33	muscle attachment / muscle development
17	CG9470	*metallothionein A*	-67	metal ion homeostasis

### Hand activity is evolutionarily conserved

**Fig 3:**
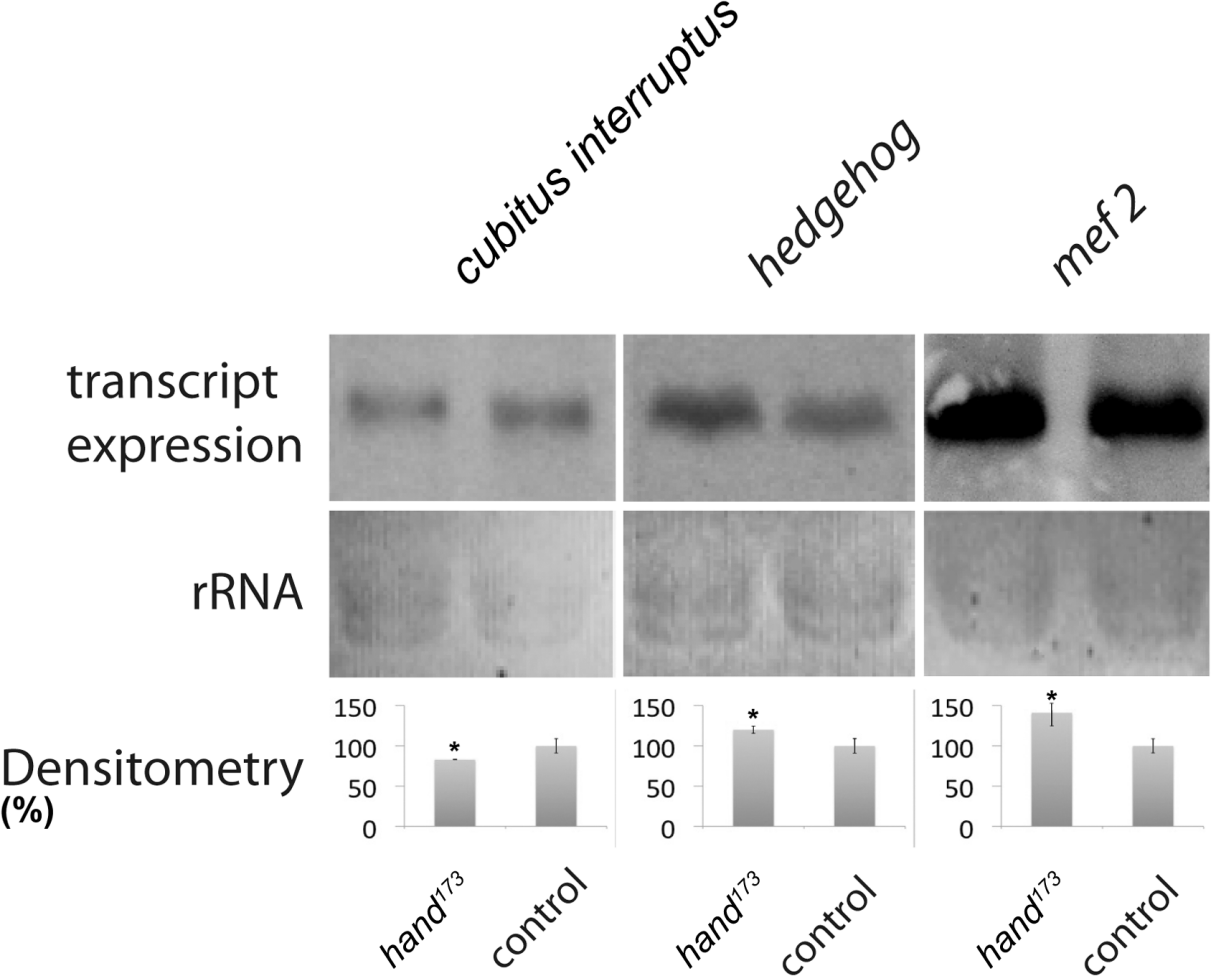
Relative expression of *Drosophila* homologs of selected vertebrate Hand target genes in *hand* mutant *Drosophila*, compared to wild type. Expression levels were assessed by Northern blot. Statistically relevant deviations are evident with respect to all genes tested. While *mef2* and *hedgehog* display an increased expression of 40.6% and 19.8% in *hand* ^*173*^ animals, respectively, the expression of *cubitus interruptus* is decreased by 19.8% in the same line. Bars represent mean values ± SD of at least three independent experiments. Asterisks indicate statistical significance (Student´s *t*-test *P*<0.05). Quantification of the relative band intensities in relation to the loading controls (ribosomal RNA, rRNA) was done by densitometric analysis.

In order to evaluate a possible evolutionary conservation of Hand activity, in addition to the genes described above we also analyzed the Hand dependent expression of *Drosophila* genes whose vertebrate homologs had previously been identified as Hand targets. Genes tested were *hedgehog*, *cubitus interruptus* and *mef2*, with the former two factors being regulated by vertebrate Hand at the transcriptional level [41, 42] and the latter one being activated at the posttranslational level [43]. The respective genes were selected independently of their microarray derived expression levels. As a result of comparative Northern blot analyses we found that all genes exhibit significantly deviant expression levels in the *hand* mutant: while *mef2* and *hedgehog* displayed an increased expression of 40.6% and 19.8%, respectively, the expression of *cubitus interruptus* was decreased by 19.8% in *hand* ^*173*^ animals, thus confirming a Hand dependent expression of all selected genes (Fig 3).

## Discussion

### Hand regulates the expression of numerous genes

Despite the fact that from embryogenesis to adulthood Hand is substantially expressed in all cells constituting the heart, animals lacking Hand expression do not exhibit any morphological abnormalities in the dorsal vessel at embryonic or larval stages. As previously shown, distinct phenotypes become apparent only later in development with adult *hand* ^*173*^ flies exhibiting severe malformations, such as disorganized myofibrils in heart and midgut tissue and a significantly reduced systolic and diastolic diameter of the heart lumen [24]. Nevertheless, the early expression of Hand in heart cells that is maintained during larval life indicates that Hand is of functional importance already in these stages of *Drosophila* development. In this context, a possible function could be the fine regulation of gene expression in the respective tissue rather than being a major transcriptional activator / repressor of factors that are essential for cardiac integrity and survival. To analyze this indication in more detail, we aimed to identify Hand target genes by making use of a microarray based approach that allowed us to examine alterations in gene expression in a genome-wide manner. As a result, we identified 545 genes that exhibited altered expression levels in a *hand* mutant background and that may thus represent target genes of Hand. Based on these data we assume that Hand is part of a gene regulatory network that comprises numerous transcription factors, which, in addition to regulating the expression of different downstream genes, control their individual expression in a mutual manner. This assumption is supported by the result that a considerable share of the identified Hand targets is apparently involved in transcriptional regulation (80 of 545, Fig 1, S1 Fig).

One key feature of bHLH proteins represents their intrinsic ability to form homo- and heterodimers with other bHLH proteins, which eventually results in a highly heterogeneous mixture of activators or repressors in different cell types. As shown previously, Hand forms heterodimers with, e.g., class I bHLH proteins such as Daughterless (Da), class II bHLH proteins such as Twist or Nautilus [26, 44–46] or class-O transcription factors such as Hey [26]. Since *hand* null mutants display only mild phenotypes that do not result in embryonic lethality, we argue that Hand acts most likely as a tissue specific modulator of transcriptional activities rather than being a key regulator such as Twist or Da. This presumption is further supported by the fact that none of the putative Hand targets identified in this study exhibited a complete knock-out as a result of Hand deprivation. In any case a considerable residual expression remained, which is different compared to master transcription factors such as Twist, whose binding to a *cis*-regulatory module represents a prerequisite to activate expression of a large percentage of its target genes [47]. This disparity indicates that, unlike Twist, Hand modulates expression of its targets in a rather subtle manner.

### Hand regulates the expression of genes that are critical to heart and muscle function

The most prominent phenotype in *hand* null mutants is a disorganized myofibrillar architecture being apparent in the dorsal vessel [24] and in wing heart muscles [26]. In the heart, the myofilaments are normally organized in a helical fashion, which allows the heart lumen to narrow upon contraction. This provides the driving force for hemolymph flow from the posterior heart chamber towards the anterior aorta portion of the heart. In *hand* null mutants the orientation of myofibers is disturbed, which likely accounts for the observed reduced systolic and diastolic diameter and abnormal heart beating [24]. Similarly, myofibrillar organization defects were found in muscle cells of the wing hearts of *hand* mutants [26]. Based on these observations, we expected Hand to be involved in regulating the expression of genes encoding proteins essential to sarcomere assembly or myofilament differentiation in general. Congruously, we found *akirin*, *kugelei*, *msp-300*, *multiplexin*, and *zasp52*, all of which known to be crucial to muscle architecture or function, being downregulated in *hand* mutant animals. While Akirin represents a critical cofactor of the key *Drosophila* mesoderm and muscle transcription factor Twist [36], Kugelei was reported to be essential for the spatial orientation of actin bundles [37]. Msp-300 is required for proper muscle function by forming a nuclear ring structure that recruits and associates with a network of polarized astral microtubules, enabling the dynamic movement and uniform spacing between the nuclei in each muscle fiber. Disruption of this mechanism considerably impairs muscle function and larval motility [48]. With respect to Multiplexin it was recently shown that the protein is required for heart morphogenesis by controlling the direction, timing, and presumably the extent of Slit/Robo activity and signaling at the luminal membrane of cardioblasts [38]. Finally, Zasp52 is a member of the PDZ-LIM domain protein family and required for muscle attachment as well as Z-disk assembly and maintenance [39]. Noteworthy, transcription of the respective genes is not completely blocked, a finding, which further substantiates the assumption that Hand participates in transcriptional fine regulation rather than being the sole regulator of these genes. Nevertheless, the simultaneous knock-down of these factors could readily account for the disorganized myofibrillar architecture observed in *hand* mutant animals.

Moreover, the complex gene structure of *zasp52* and *msp-300* with several splice variants present (http://flybase.org/) raised the question whether Hand is also involved in regulating expression of these genes in a splice variant specific manner. At least with respect to *zasp52* our data indicate such a regulation. As depicted in Fig 2, Northern blot analysis revealed the expression of two major *zasp52* transcripts in 3^rd^ instar larvae with the larger splice variant being downregulated by 44.8% in the *hand* mutant and the smaller one being upregulated by 34.9% in the same line. An estimation of the respective molecular masses yielded that the larger transcript consists of about 4700 nucleotides, while the smaller one comprises about 3100 nucleotides (S1 Fig). According to sequence predictions (http://flybase.org/), except for isoform Q the applied riboprobes should detect all 17 remaining *zasp52* splice variants ranging in length between 951 nucleotides (splice variant P) and 7418 nucleotides (splice variant F). Apparently, only two of them are expressed in considerable amounts in 3^rd^ instar larvae (Fig 2, S1 Fig) with the corresponding size of the detected transcripts rendering it likely that the larger one represents either isoform I (4649 nucleotides), M (4790 nucleotides), or N (4637 nucleotides), while the smaller one presumably corresponds to either isoform E (3176 nucleotides), R (3002 nucleotides), S (3197 nucleotides), or U (2984 nucleotides), respectively. To unambiguously identify the respective transcript variants, clearly additional Northern blots with isoform-specific riboprobes are necessary; however, such an analysis was not the focus of the present study.

By providing evidence that the expression of distinct factors critical to muscle integrity and function is regulated by Hand, this work represents a promising starting point for future studies aiming to understand the physiology of distinct cardiac phenotypes that manifest in Hand mutant animals [24, 26]. Based on our data, further research will clarify whether or not impaired expression of the identified factors, either exclusively or in a concerted manner, is responsible for the respective phenotypes. Taking into account that Hand activity appears to be evolutionarily conserved, appreciation of Hand functionality in *Drosophila* may be highly beneficial with respect to understanding the physiological relevance of vertebrate Hand in a more complete manner.

## Supporting Information

S1 Fig Isoform specific expression of *zasp52* analyzed by Northern blot. In both, 3rd instar larvae of *hand* mutant animals (*hand* ^*173*^) as well as wild type animals (control), two major transcripts are detected. The larger one (white arrow) migrates at about 4.7 kilobases while the smaller one has a length of about 3.1 kilobases (black arrow). MWM: molecular weight marker.
(TIF)

S1 Table Classification of genes exhibiting deviant expression levels in *hand* ^*173*^ animals. Analysis of the microarray data yielded 545 genes exhibiting an altered expression in *hand* ^*173*^ hearts, with 385 genes being downregulated in the mutant and 160 genes displaying an increased expression in the same line, compared to wild type. Classification of the corresponding protein products into functional groups was done manually utilizing data from NCBI (http://www.ncbi.nlm.nih.gov/) and Flybase (http://flybase.org/). Factors with yet unknown physiological functions are allocated to the category “assorted other”.
(XLSX)

S2 Table qRT-PCR Primers. (DOCX)
