# Supplementary figures and images for: The bHLH Transcription Factor Hand Regulates the Expression of Genes Critical to Heart and Muscle Function in Drosophila melanogaster
Source: PLoS One. 2015 Aug 7;10(8):e0134204. doi: 10.1371/journal.pone.0134204 (PMC4529270; doi:10.1371/journal.pone.0134204)

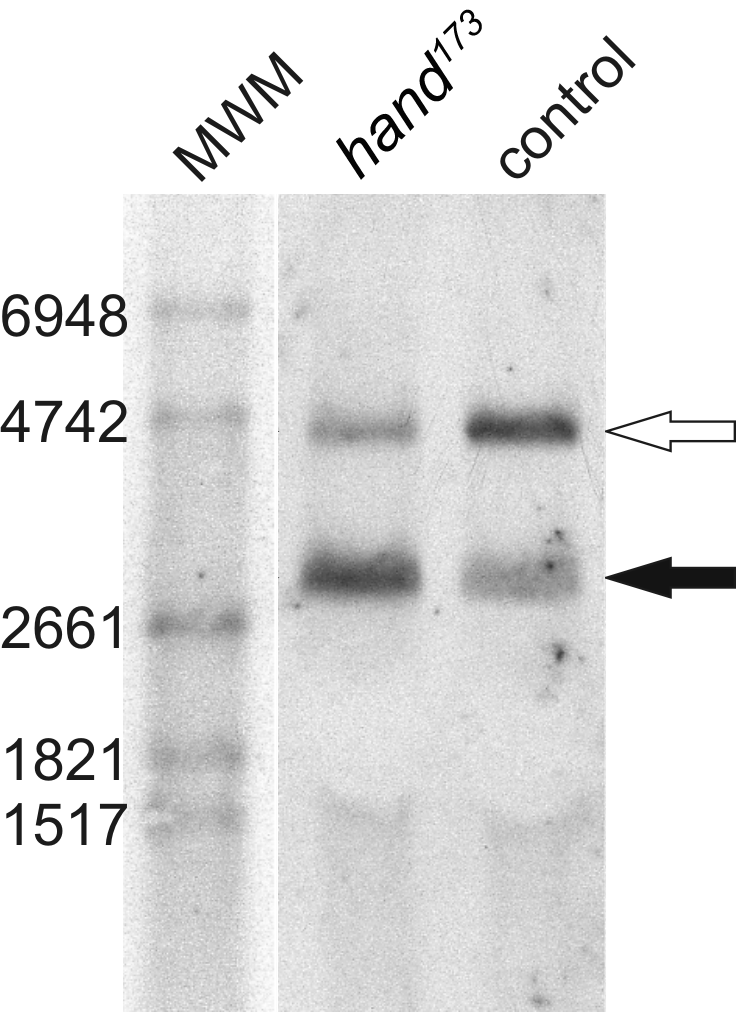

Supplement: S1 Fig — In both, 3rd instar larvae of hand mutant animals (hand 173) as well as wild type animals (control), two major transcripts are detected. The larger one (white arrow) migrates at about 4.7 kilobases while the smaller one has a length of about 3.1 kilobases (black arrow). MWM: molecular weight marker. (TIF) [file pone.0134204.s001.tif]
