# Supplementary material for: The bHLH Transcription Factor Hand Regulates the Expression of Genes Critical to Heart and Muscle Function in Drosophila melanogaster
Source: PLoS One. 2015 Aug 7;10(8):e0134204. doi: 10.1371/journal.pone.0134204 (PMC4529270; doi:10.1371/journal.pone.0134204)
Supplement: S2 Table — (DOCX) [file pone.0134204.s003.docx]

**Supporting Table 2**

*Primers used for qRT-PCR*

| CG1161.FWD | [AGGAGCACACCAACGAAGATGATG](about:blank) |
| --- | --- |
| CG1161.REV | [CCACACGATTGAGGACATTGGC](about:blank) |
| CG1429.FWD | [ATGAACGCAATCGGCAGGTGAC](about:blank) |
| CG1429.REV | [GCGTACTGGTACAGCTTGTTGC](about:blank) |
| CG1520.FWD | [TGCTTACCTTCGAGGGCAGTGATG](about:blank) |
| CG1520.REV | [TGCCGGTTGCGTTTCTCTTGTC](about:blank) |
| CG1844.FWD | GGTTTGCCATTAAGCAGCTATTC |
| CG1844.REV | GCCTGGTTATTGTTGCCCGTAAAG |
| CG2145.FWD | [AAGGAGCAGGACATTGGCACGAAG](about:blank) |
| CG2145.REV | [CTGGCTTATTGAGTCCCTGATGCG](about:blank) |
| CG2125.FWD | [TGTGCATATGCGGCGTCATACC](about:blank) |
| CG2125.REV | [TGCCTTAAAGCAGCCTTCAAACG](about:blank) |
| CG2233.FWD | [ACACAACTCGCCCTTCTATCGC](about:blank) |
| CG2233.REV | [TGTTCCTCAGCCAGGACACTTG](about:blank) |
| CG3186.FWD | TGAAGGTTCCAGAGGGCGAATTG |
| CG3186.REV | GAGAACGGTGACAGTAAGTCC |
| CG3869.FWD | [AGCGCCTCTCGTGGACAAATTC](about:blank) |
| CG3869.REV | [GCAAAGGTGCTCGACAATTCCTG](about:blank) |
| CG4637.FWD | [ATCGCGACCAGTCCAAATACGG](about:blank) |
| CG4637.REV | [TGGGAACTGATCGACGAATCTGAC](about:blank) |
| CG4699.FWD | CGCTGACGAGATGGTAACCTA |
| CG4699.REV | GGGCAATGGCTGCACGATCTC |
| CG4716.FWD | [ACAACACCAGCTGCGGTAGAAG](about:blank) |
| CG4716.REV | [ATCCTCCGTGCTTCTGCGTTTC](about:blank) |
| CG5277.FWD | [CAAAGCGAAAGGCATGGAAGCG](about:blank) |
| CG5277.REV | [GAACTTTGGTGGCTTGCGTGTG](about:blank) |
| CG5320.FWD | [TCGCCTGACCTTCAAGTATGAGC](about:blank) |
| CG5320.REV | [TTGACTGTTGGACTGAGGCAAG](about:blank) |
| CG5399.FWD | [ATGATGCCTCCGTGTCCTTCAG](about:blank) |
| CG5399.REV | [AACTGGCACGGCAATCCAACTG](about:blank) |
| CG6090.FWD | [TCCAACGCTTGACTCTGAGACG](about:blank) |
| CG6090.REV | [CACAATGCGCCTCTTGTTGGAG](about:blank) |
| CG6105.FWD | [TTGGCTACCAAGGGATCAGGAC](about:blank) |
| CG6105.REV | [TTCAGGAACACGTCCAGTTGGG](about:blank) |
| CG6746.FWD | [AGGCCAACTGAACCTTGACCAG](about:blank) |
| CG6746.REV | [AGTGCGACGGCCAATGATATGC](about:blank) |
| CG7749.FWD | [TCGACAACTGGCGCTATTGAACTG](about:blank) |
| CG7749.REV | [TGGGCTTTCCCATATCACTAACCG](about:blank) |
| CG8226.FWD | [CGAGGGAGTTAAGGATCGTTTGGG](about:blank) |
| CG8226.REV | [GGCTCAGCTCCCTTCATAAATCCC](about:blank) |
| CG8369.FWD | [GTGCGTCATGGCCAACTACAAC](about:blank) |
| CG8369.REV | [CCTTGGACTTCTGCTCGAATGG](about:blank) |
| CG8580.FWD | [AGGAGCGCGAGAATCAGCTAAG](about:blank) |
| CG8580.REV | [GTACACAGCTTTACGACAGGTAGC](about:blank) |
| CG9470.FWD | [ACTGCGGATCTGACTGCAAGTG](about:blank) |
| CG9470.REV | [AGACAAGATGCAGCGCCTCTAC](about:blank) |
| CG10484.FWD | [CTCGAAAGAGTCGCGCTTCATC](about:blank) |
| CG10484.REV | [TTCAGCTTGCGACGAGTGTTGG](about:blank) |
| CG10679.FWD | [TCTCCGGCAAGCAAATGAATGAC](about:blank) |
| CG10679.REV | [TGAAGAACGGATCCACCTTGCAC](about:blank) |
| CG10811.FWD | [CCTACACGGCATACTGTTAAAGCC](about:blank) |
| CG10811.REV | [TGCTACAGGAGCAACAGAACGG](about:blank) |
| CG11271.FWD | [ACAATGGCCGACGTTGATGTTG](about:blank) |
| CG11271.REV | [TCCTGGAGGGCAGTGTTAATGTC](about:blank) |
| CG11525.FWD | [TCCTGCAGCAGTACATGAGGATTC](about:blank) |
| CG11525.REV | [GGGACAGAATACCCGAAACGATGC](about:blank) |
| CG11661.FWD | [ACCAGATCTTCACCCAGTTCGC](about:blank) |
| CG11661.REV | [GTACTTGACATCACCAGAGCCATC](about:blank) |
| CG12400.FWD | [CGGCACTACATCGAACTGCATC](about:blank) |
| CG12400.REV | [GCCATAGGTCTTGCGTTCCTTC](about:blank) |
| CG14616.FWD | [AGCTCGTCCGCTCCAAATCTTC](about:blank) |
| CG14616.REV | [TGGTGGTACATCGCCAAATCCG](about:blank) |
| CG15067.FWD | AAGGTGACCATCAACGGCAAGTG |
| CG15067.REV | TGTGCGTGGTGGTAGTCGTTTG |
| CG16713.FWD | TGACGCACTCGTTTCGATCGG |
| CG16713.REV | ACGCCTTGGCCCTGAAAAATG |
| CG16747.FWD | [TTCGACTCGATTTCTTCTCAGTGC](about:blank) |
| CG16747.REV | [GCCACTACTAAACACTGGGTCGAG](about:blank) |
| CG17108.FWD | [ATGGATCTGGTGGTCACGGTTCTG](about:blank) |
| CG17108.REV | [CCAAGTCCTAAATCACCGCCAGAG](about:blank) |
| CG17820.FWD | ACTGCGTTAGGCGTTCGATTGG |
| CG17820.REV | TCCATTCGCCTGGCTCATTTGG |
| CG18039.FWD | [TGGTTCACAACGGGAGCGTTACTG](about:blank) |
| CG18039.REV | [ATTGTCCGCGTGCTGAGAGCTTTG](about:blank) |
| CG18107.FWD | [TGCAATCGTCACTGTCTTTGTGC](about:blank) |
| CG18107.REV | [GGGTGACAACGGAATAGCATTGG](about:blank) |
| CG30084.FWD | [AATGCGAGCGCCTCATTACTGG](about:blank) |
| CG30084.REV | [TGGCACACTTGAAGCACTCCAC](about:blank) |
| CG30415.FWD | TCTGGCGCTACTATTCATTGCG |
| CG30415.REV | GTTGGCCAGCTTGCTGATCTTG |
| CG31509.FWD | [TTCCGGTTTGCTTCAGCGTTCC](about:blank) |
| CG31509.REV | [AGCAGCAGTGCAAAGCACATAAG](about:blank) |
| CG33171.FWD | [CAGGATCGCAAACGCAAGAAGC](about:blank) |
| CG33171.REV | [TTCACTTTCGCCGTGACTGCTG](about:blank) |
| CG33256.FWD | [TGAAGAGCCCAATGGAGGATCG](about:blank) |
| CG33256.REV | [TCTCCCACAAAGGCAAGACTGC](about:blank) |
